# Some Unresolved Ethical Challenges in Healthcare Decision-Making: Navigating Family Involvement

**DOI:** 10.1007/s41649-020-00111-9

**Published:** 2020-03-05

**Authors:** Sumytra Menon, Vikki A. Entwistle, Alastair V. Campbell, Johannes J. M. van Delden

**Affiliations:** 1 Centre for Biomedical Ethics, Yong Loo Lin School of Medicine, National University of Singapore, Singapore; 2 Julius Center for Health Sciences and Primary Care, University Medical Center Utrecht, Utrecht, Netherlands

**Keywords:** Decision-making, Relational autonomy, Family involvement, Competent patient

## Abstract

Family involvement in healthcare decision-making for competent patients occurs to varying degrees in many communities around the world. There are different attitudes about who should make treatment decisions, how and why. Legal and professional ethics codes in most jurisdictions reflect and support the idea that competent patients should be enabled to make their own treatment decisions, even if others, including their healthcare professionals, disagree with them. This way of thinking contrasts with some cultural norms that put more emphasis on the family as a decision-making entity, in some circumstances to the exclusion of a competent patient. Possible tensions may arise between various combinations of patient, family members and healthcare professionals, and healthcare professionals must tread a careful path in navigating family involvement in the decision-making process. These tensions may be about differences of opinion about which treatment option is best and/or on who should have a say or influence in the decision-making process. While some relevant cultural, legal and policy considerations vary from community to community, there are ethical issues that healthcare professionals need to grapple with in balancing the laws and professional codes on decision-making and the ethical principle of respecting patients and their autonomy. This paper will highlight and propose that a partial resolution to these issues may lie in relational understandings of autonomy, which in principle justify interventions by healthcare professionals and family that support patients in decision-making.

## Introduction

In most jurisdictions, legislation and professional ethical codes emphasize the right of competent patients to decide between treatment options for themselves, even if their decisions differ from the doctor’s recommendations or go against the advice of caring family members. This legal and regulatory emphasis reflects much bioethical thinking about decision-making that has emphasized personal autonomy and in a way that highlights individual choice and independence (Beauchamp and Childress 2013, 459; Beauchamp 2011). Another way of thinking, shaped by Confucian principles, focuses on the family rather than the patient as the primary decision-making entity.

We will use experiences from Singapore to analyse family involvement in decision-making for competent patients, in light of the cultural norms and legal and policy context. The population in Singapore and its South East Asian neighbours is culturally diverse, with a mixture of eastern and southern Asian, as well as western cultural influences, and therefore varying attitudes about who should make treatment decisions (Tao 2000). Nevertheless, the ethnic majority in Singapore are of Chinese descent, and the strong influence of Confucian principles has meant that family involvement in treatment decision-making is rather pervasive, although the extent, type and whether the involvement is supportive of the patient having a say vary (Chia 2011; Koon and Krishna 2014). Despite the Confucian influences in Singapore, competent patients have the legal right to make their own treatment decisions, and the professional codes for doctors reflect the same right (Re LP 2006; Singapore Medical Council 2016a). In practice there are some examples of decisions being made in cooperative harmony involving the patient, family and healthcare professionals, and other cases, particularly when patients have been diagnosed with a serious illness or are nearing end of life, where patients may be excluded from decisions, and sometimes family members may not agree (Tai 1997; Fan 1997).

Significant family involvement in treatment decision-making for competent patients contrasts with (although it need not be incompatible with) laws and professional codes that focus on individual rather than family rights to decide. There are particular tensions and challenges around family involvement where healthcare professionals and patients may wish to give more weight to family perspectives than the law and professional codes allow (Singapore Medical Council 2016b). This raises questions about how family involvement should be navigated and accommodated by healthcare professionals while complying with laws and professional codes on decision-making, with an ethical principle of respecting patients and their autonomy.

This paper should be of interest beyond Singapore and South East Asia because family involvement in healthcare decision-making for competent patients occurs everywhere, and although some of the cultural, legal and policy considerations may be different, we highlight issues that many healthcare professionals need to navigate. We propose that relational understandings of personal autonomy may offer a partial resolution to some of these issues.

## Healthcare Decision-Making in Singapore

We will first consider how healthcare decision-making is practiced in Singapore before reviewing the relevant legal framework and professional codes and analysing practice in this context.

### Family Involvement in Practice

There is evidence of significant family involvement in healthcare decision-making in Singapore and of patients’ autonomy sometimes being limited by professional deference to family preferences. The practice of collusion, where the healthcare professionals and relatives agree to hide the truth about the diagnosis and/or prognosis from patients, has been common, driven primarily by a desire to “protect” patients from bad news (Goh 2008a, b; Tan et al. 1993). One retrospective review of records of patients who died in a hospital oncology ward found that 6 out of 51 patients classified as “alert” (interpreted to mean competent at the relevant time) were not informed of their diagnosis within 28 days of diagnosis. At the time of initiation of a do not attempt resuscitation order, 32 patients were alert, but only 3 were consulted about treatment decisions relating to end of life care, whereas the families were involved in all of them. Older patients and those who did not speak English were less likely to be involved in treatment decision-making (Chong et al. 2015).

Another study used a short video prompt to help investigate the attitudes and perspectives of cancer patients and family caregivers to treatment decision-making (Tay et al. 2017). The video featured a woman in her 60s who was in hospital for medical tests. Her three adult children learned the test results and so found out that their mother had advanced cancer, when they looked at her medical file at the nurses’ station when no one was around. On the video, the adult children expressed different views about whether to reveal the diagnosis to their mother. The overwhelming majority of study participants (129 out of 132 patients and caregivers) thought that patients should be told their diagnosis, emphasizing the importance of their right to know. The study findings also indicated greater family involvement in treatment decision-making when patients are elderly and have poor health, poor prognosis, poor anticipated response to treatment and metastatic disease.

While the video prompt study showed that most patients and caregivers want to know their diagnosis, the record-based study discussed above revealed that 11.7% of patients were not informed of their diagnosis. This suggests that a gap between what people think is the ideal and actual practice.

Overall, these studies indicate that families wish to “protect” patients from bad news relating to a diagnosis and/or prognosis and are more involved in decision-making for elderly patients, especially as they are approaching the end of their lives.

### Law, Professional Codes and Policy

The legal position in Singapore is currently based on case law (there is currently no statutory law on this issue). In the case of Re LP (2006), the Singapore High Court held that competent persons have the right to make their own healthcare decisions. This right is also reflected in the Ethical Code and Ethical Guidelines and the accompanying Handbook on Medical Ethics (Handbook), produced by the professional regulatory body, the Singapore Medical Council (2016a, b). The legal and professional positions in Singapore are similar to those in many jurisdictions around the world including the UK, the USA, the Netherlands and Canada. They developed, in part, to protect patients from being unduly influenced by their doctors’ preferences and interests. In Singapore, the “right not to know” was upheld by the Court of Appeal in the Hii Chii Kok v Ooi Peng Jin London Lucien (2017) case, which determined that competent patients could waive their rights to be informed of relevant information about treatment, or alternative treatment options, if they insist on not being informed about it and if doctors have explained the serious consequences of their decision (Hii Chii Kok v Ooi Peng Jin London Lucien 2017). Menon and Voo (2018) noted that the requirements placed on doctors to be satisfied that patients appreciate those consequences may prevent the waiver from being misused.

In a section on collusion, the Handbook suggests that doctors who are asked by families to withhold information from patients should advise that withholding information from patients is usually inadvisable and they should reassure families that any disclosure to patients will be managed sensitively (Singapore Medical Council 2016b). However, the Handbook also recognizes the importance of families, and in a section on providing relevant information to patients, it suggests that doctors may consult with families, colleagues or an Ethics Committee when considering balancing the benefits and harms of delaying disclosure to patients (Singapore Medical Council 2016b). However, it cautions that doctors should be mindful that some patients may not want their families to know their medical information, and in those cases, patient confidentiality should be maintained (Singapore Medical Council 2016b). Similarly, the Handbook acknowledges the potential value of family presence in medical consultations but cautions doctors against assuming that the family have a right to be present because relationships between the patient and family may be complicated (Singapore Medical Council 2016b). It recommends that family could attend consultations if the patient agrees, if there is no evidence that the patient has been coerced or unduly pressured by the family to permit them to attend and if the doctor does not believe that family presence will disrupt the doctor-patient relationship (Singapore Medical Council 2016b).

While the law and professional guidance in Singapore emphasize individuals’ authority to make treatment decisions, Confucian-inspired policies reinforce familial responsibilities with respect to caregiving and paying for healthcare costs (Chin and Phua 2016; Teo et al. 2006, 180). Three examples of these policies illustrate this: first, the government has actively encouraged families to care for their elderly relatives by offering priority public housing, subsidies and tax incentives to adult children who live with or near to their parents (Chan 1997; Frankenberg et al. 2002). Second, the Maintenance of Parents Act, Cap 167B 1996 requires adult children to provide funds to maintain the basic needs, including medical costs, of their parents aged 60 and over, if the parents are unable to provide for themselves (Maintenance of Parents Act, Cap 167B 1996). Third, the Medisave account in the compulsory national social security savings scheme, called the Central Provident Fund (CPF), is primarily for healthcare needs and may be used to pay for current or future selected healthcare expenses of CPF members and their immediate family members (spouse, parents, grandparents and children).

### Analysis

The significant family involvement in healthcare decision-making for serious illnesses in Singapore in part reflects the Confucian practice of filial piety, which manifests in behaviours aimed at protecting and supporting sick and ageing parents in their various vulnerabilities as patients. It does, however, seem that collusion is not as acceptable as it once was and people want to know about their healthcare conditions and be involved in decision-making rather than being kept in the dark (Low et al. 2009). Although most instances of collusion are contrary to the law and professional codes, they occur to varying degrees because cultural and family norms deem it acceptable.

Some key issues and dimensions of family involvement were elucidated in the TRIO study, which conceptualized the triadic nature of decision-making involving the clinician, caregiver and patient.

The authors recognized that many factors, such as demographic, cultural and medical factors, can shape family involvement throughout the decision-making process (Laidsaar-Powell et al. 2017). The TRIO framework focused on the key caregiver and acknowledged but did not examine situations where there are multiple caregivers involved in treatment decision-making, which may occur in some families. The TRIO study generated two sets of guidelines for cancer settings, one on facilitating effective family involvement and one on managing challenging family interactions (Laidsaar-Powell et al. 2018a, b). The latter recommended strategies on managing challenging situations, such as undue family influence, and conflicts between patients and families regarding treatment decisions, which occur in all communities, including Singapore. As the authors themselves noted, these guidelines and strategies were crafted primarily by experts living in and based on studies conducted in Western nations. This has not been explored in communities with different cultural and/or policy norms. Further research is needed to study these issues.

There is evidence in the cancer and geriatric primary care settings to show that family involvement in treatment decision-making may have positive effects, such as lowering patients’ anxiety, improving communication and enhancing patients’ relationship with the doctor, and negative effects, such as pressuring patients through controlling behaviour and inundating patients with too much information (Öhlén et al. 2006; Clayman et al. 2005; Shin et al. 2017). Furthermore, studies have revealed that patients, family and healthcare professionals generally value family involvement in treatment decision-making. Adult children in countries with less explicitly family-centric decision-making culture, such as the Netherlands, also often want to take care of their parents (although not to be coerced into doing so) (Stuifbergen et al. 2010). In South Korea, Shin et al. surveyed 1450 cancer patients and caregivers (725 each) and reported that 65.65% of patients and 67.58% of caregivers preferred that patients and families made treatment decisions together, whereas 14.3% of patients and 14.2% of caregivers preferred that patients made decisions after considering family views (Shin et al. 2017). In Germany, Schäfer et al. (2006) found that 89% of 50 patients and their relatives thought that healthcare decisions should be made jointly between patients, family and clinicians. In Australia, many cancer patients, family members, nurses and doctors thought that the family should be involved because the decisions affected them and their involvement could positively enhance patients’ autonomy (Laidsaar-Powell et al. 2016). In the latter two studies, most participants thought that the competent patients were the locus of authority even though the families were involved in the decision-making process. In the Australian study, a few patients and family members were satisfied with family dominance in treatment decision-making, although the clinicians were apprehensive about it (Laidsaar-Powell et al. 2016).

In many countries then, family involvement in treatment decision-making can generate a tension in interactions between patients, family and healthcare professionals. On the one hand, law and professional codes emphasize the need for patients to be informed and enabled to decide for themselves and give informed consent for treatment. On the other hand, withholding relevant information about treatment from patients and having other family members lead on decision-making are the cultural preferences within some families. This raises the question of how family involvement may be appropriately accommodated by healthcare professionals while maintaining consistency with professional codes and the law. We suggest that relational understandings of autonomy could be used to generate some guidance on how this accommodation may be achieved.

## Relational Understandings of Autonomy may Offer a Way Forward

Autonomy refers to the capacity of people to govern themselves, make choices and define outcomes (Stoljar 2011). Various theories have been developed over the past decades about the meaning of respecting someone’s autonomy, and it is increasingly recognized that autonomy is relational (Mackenzie and Stoljar 2000, 328).

Relational conceptions of autonomy recognize that people and their capacity for or exercise of autonomy are shaped by their interpersonal relationships and broader social conditions, including cultural and societal norms (Ells et al. 2011). This contrasts with the emphasis on respect for autonomous choices which features in much medical literature and which at least implicitly idealizes independence and individual choice (Beauchamp and Childress 2013, 459; Beauchamp 2011). The consideration of interpersonal relationships and broader social conditions in relational conceptions of autonomy stresses that people are interdependent (Mackenzie and Stoljar 2000, 328). Relational accounts recognize relationships, involving family and community, as key to how people gain their values, decision-making capabilities, identities and aspirations or life plans and thus to how they develop and express autonomy (Dove et al. 2017). On relational accounts, someone’s capability to exercise autonomy is dynamic and can be fostered or undermined by features of social situations and relationships and the way the person is positioned within them. In medical contexts, relational accounts highlight the potential significance for autonomy of professional-patient relationships and expressions and experiences of respect and trust, as well as opportunities to ask questions and discuss concerns about potential treatment options (Dodds 2000; Entwistle et al. 2010).

In treatment decision-making, there can be tensions between respecting competent patients’ autonomy while accommodating family interests. While there is no clear boundary to indicate when family involvement strays from appropriate and supportive to inappropriate and stifling, relational thinking draws attention to how family and healthcare professional involvement can either foster or diminish patient autonomy (Durocher et al. 2018). Anita Ho (2008) proposed a solution for addressing undue family pressure in an attempt to balance the patients’ interests with those of the family. She suggested that clinicians concerned about family pressure should conduct private discussions with patients to understand how the treatment decision was reached in light of the patients’ goals and preferences. Ho acknowledged that clinicians may lack the necessary skills and knowledge about family dynamics to navigate potential family pressure, and so instances of inappropriate interference may not always be identified and/or resisted. Roy Gilbar, who studied family involvement in decision-making in a general practice setting, also noted that while many doctors were willing to form alliances with the family to persuade patients to accept their advice, they struggled when family had different moral principles or different agendas from their own or that of the patients’ (Gilbar 2012). Gilbar cautioned doctors against forming too strong an alliance with a patient’s family because the discomfort and pressure from a strong alliance could undermine the patient’s autonomy (Gilbar 2012). Regarding family pressure, most doctors preferred a non-confrontational approach involving mediation and discussions to explore family and patients’ concerns. Ho and Gilbar both illustrate that relational thinking can aid in distinguishing between appropriate and inappropriate family involvement in treatment decision-making but finding a resolution when tensions surface is more complex.

Our view is that relational accounts of autonomy can help justify supportive interventions by the healthcare professional and family as long as they view decisional authority as located primarily with the patient and serve to enable the patient to exercise their decision-making authority in an authentic way. Relational accounts of autonomy, like the accounts of autonomy that emphasize independence and choice, will not justify forms of collusion that impede patients’ understanding of their situation and scope to influence decisions that affect them.

## Preserving Autonomy-Supporting Family Involvement while Complying with the Relevant Laws and Professional Codes

It is challenging to ascertain how to facilitate the autonomy-supporting family involvement that many patients and healthcare professionals welcome in treatment decision-making while complying with laws and professional codes that emphasize the patient as the authorized decision-maker.

In Singapore, the Handbook offers doctors some flexibility, which is not reflected in the law, to legitimately involve families in scenarios involving information disclosure when there is concern for a patient’s wellbeing while balancing the benefits and harms and considering the duty to maintain confidentiality. The recognition in the Handbook of the important role family plays in these situations is important and reflects more of the reality of clinical practice, especially but not only in Singapore (Singapore Medical Council 2016b). The Handbook’s guidance on approaching families who seek collusion with a view to convincing them that patients should be informed of the relevant information is ethically sound and reinforces the legal stance that most instances of collusion are unacceptable. Furthermore, the recognition of coercion and family pressure in the Handbook alerts doctors to be mindful of family dynamics and the role it can play in the healthcare setting. The guidance in the Handbook can be interpreted as congruent with relational accounts of autonomy that justify supportive family involvement while respecting the patient. This way of thinking can help doctors and other healthcare professionals ascertain in particular cases whether the “right” balance has been struck.

However, the Confucian-influenced social and healthcare financing policies in Singapore have inculcated familial responsibility to care for relatives and pay their healthcare costs. They have also engendered within families an expectation and a sense of entitlement to be involved in treatment decision-making, since the patients’ decisions will likely have a significant impact on them. It may be a sensitive task for the doctor to ascertain patients’ preferences about the extent of family involvement when the family, the healthcare professional and/or the patient may think they are entitled to be involved somehow. Nonetheless, this is vital information for the navigation of treatment decision-making.

We tend to agree with Gilbar’s proposal that the legal and professional model should explicitly provide scope for family involvement in decision-making processes but that it should also ensure the patient retains the locus of authority (Gilbar 2011). Family practices such as helping a patient to understand medical information, listening to concerns and providing emotional support can all be consistent with this. We recognize that for some families or particular family members, the responsibility to care for relatives, pay for their treatments and be involved in treatment decision-making may be significantly burdensome, particularly if they have tight financial constraints, pressures at work and young children to care for. The full details of how and to what extent families should be involved in the decision-making process will be to at least some extent dependent on the specifics of the situation (including family relationships). There is no scope to develop a fuller account in this paper, but the basic idea is that the involvement of both healthcare professionals and any relevant family members should be autonomy-supportive rather than autonomy-undermining. An orientation to this idea should help preserve positive family involvement, discourage the formation of strong alliances between health professionals and family members that render the patient fearful or unable to have their say and avoid the exertion of undue pressure on patients to make particular decisions. It should help ensure that practice complies with the relevant laws and professional codes.

## Conclusion

Healthcare professionals have a vital role in decision-making and have a responsibility to promote their patients’ autonomy and navigate the tensions that may emerge when families become involved in the decision-making process. Family involvement is widely valued by patients and can serve among other things to enhance patients’ autonomy. It can sometimes, however, be problematic, and health professionals need to be alert to this. Relational accounts of autonomy can help guide assessments of the appropriateness or otherwise of different forms of family involvement (and health professionals’ facilitation of) in treatment decision-making.
